# A novel *Streptomyces* spp. integration vector derived from the *S. venezuelae* phage, SV1

**DOI:** 10.1186/1472-6750-14-51

**Published:** 2014-05-30

**Authors:** Bahgat Fayed, Ellen Younger, Gabrielle Taylor, Margaret C M Smith

**Affiliations:** 1 Department of Biology, University of York, York YO10 5DD, UK; 2 School of Medical Sciences, Institute of Medical Sciences, University of Aberdeen, Aberdeen AB25 2ZD, UK

**Keywords:** *Streptomyces*, Cloning, Integration vector, Serine integrase, Bacteriophage, SV1

## Abstract

**Background:**

Integrating vectors based on the *int/attP* loci of temperate phages are convenient and used widely, particularly for cloning genes in *Streptomyces* spp.

**Results:**

We have constructed and tested a novel integrating vector based on *g27,* encoding integrase, and *attP* site from the phage, SV1. This plasmid, pBF3 integrates efficiently in *S. coelicolor* and *S. lividans* but surprisingly fails to generate stable integrants in *S. venezuelae*, the natural host for phage SV1.

**Conclusion:**

pBF3 promises to be a useful addition to the range of integrating vectors currently available for *Streptomyces* molecular genetics.

## Background

Bacteria in the genus *Streptomyces* are a prolific source of natural products, many of which are used in the clinic as antibiotic, anticancer, immune-modulatory or other therapeutic agents. Furthermore these soil bacteria have an unusual life style; vegetative growth is mycelial and when nutrients become scarce a sporulation cycle initiates [1]. The phages that infect these bacteria have been exploited in the development of vectors for genetic engineering of *Streptomyces* and closely related genera, in particular in the study of natural product pathways [2].

The development of integrating vectors that integrate via site-specific recombination between a site on the plasmid vector, the *attP* site and a site in the bacterial chromosome, the *attB* site have been widely adopted by researchers wishing to genetically manipulate *Streptomyces* genes [3]. The *int/attP* site from the integrating plasmid, pSAM2, was first exploited in a novel vector that could integrate into the endogenous *attB* site in the *Streptomyces* genome [4]. The advantage of the integration vectors over freely replicating plasmid vectors are the very low copy number (usually single or two copies integrated in tandem), the ease of construction of plasmids, which can be done in *E. coli*, and the simple method of plasmid transfer into *Streptomyces* via conjugation [5].

The idea of using phage integrases by the research group at Eli Lilley, led to the development of integrating vectors encoding the *int/attP* locus from the *Streptomyces* phage ϕC31 [6-8]. The recombination event that leads to phage integration is a conservative reciprocal DNA cleavage and rejoining mechanism occurring at the centre of the *attP* and *attB* sites producing the integrated plasmid flanked by hybrid *attP/B* sites called *attL* and *attR*[7]. Phage integrases are known to be highly directional, with tight control over integration versus excision. Integration (or *attB x attP*) is the default reaction for phage integrases whilst excision (*attL x attR*) requires activation by a recombination directionality factor (RDF) or Xis [9,10]. Consequently the integration vectors based on the ϕC31 *int/attP* system and lacking any other phage genes are 100% stable in most *Streptomyces* species. The ϕC31 integrating vectors integrated with higher efficiency and were more stable than the pSAM2-derived integration vectors and are now widely adopted by researchers in *Streptomyces* genetics. The use of the ϕC31 integration system is also being widely adopted for genome engineering in eukaryotes, in particular in tissue culture and model organisms such as the mouse and *Drosophila*[11].

In 2003 the *int/attP* locus from the phage ϕBT1 was used to generate an alternative suite of phage-derived integration vectors for *Streptomyces*[12]. These vectors were demonstrated to be completely orthogonal to the ϕC31 derived plasmids and plasmids derived from the two phage *int/attP* loci could be used in combination without loss of integrating efficiency. The ϕBT1 *int/attP* integration vectors are also widely used in the *Streptomyces* community. Recently vectors based on phage TG1 have been developed for use in *Streptomyces avermitilis*[13].

Here we present a new integrating vector derived from *S. venezuelae* phage SV1 [14,15]. We demonstrate the efficient integration of the plasmid into several *Streptomyces* spp. although, surprisingly, we failed to obtain stable integrants in *S. venezuelae*.

## Results and discussion

The genome of phage SV1 was sequenced previously and, consistent with the temperate nature of the phage, *g27* encodes an integrase [15]. Gp27 is a serine integrase, whose closest homologue in the database is from *Streptomyces prunicolor* (WP_019054986.1; 54% identity). SV1 is only distantly related (between 11 and 13% identical) to ϕC31, TG1 and ϕBT1 integrases so the SV1 integration system encoded by SV1 *g27/attP* is therefore very likely to be another orthologous system to ϕC31 and ϕBT1 integration systems.

The DNA upstream and downstream of SV1 *g27* was studied for a likely *attP* site. Precedent dictates that *attP* is normally upstream or downstream of the integrase gene but in some mobile genetic elements, such as *SCCmec*, can be located quite distal from their cognate integrase genes [11,16]. In SV1 the *attP* site is unlikely to be upstream of *g27* as the upstream gene, *g28,* overlaps with *g27* by the sequence 5′ATGA, which couples the start codon (ATG) of *g27* with the stop codon of *g28* (TGA). Downstream of *g27* is a non-coding region of 342 bp before the start of the downstream gene, *g26*. The *attP* sites for the serine integrases commonly comprise a perfect inverted repeat flanking a spacer of at least 20 bp [11]. Within the *g26-g27* intergenic region in SV1 there are two perfect inverted repeats (IRs); the IR distal to *g27* has a spacer of 5 bp and the IR proximal to *g27* has a 22 bp spacer. Moreover the IR proximal to *g27* has a 10 bp perfect inverted repeat so, together with the spacer DNA, the length of this DNA element is 42 bp which is the same length as the ϕC31 *attP* site. The length *attP* sites used by other serine integrases is between 42 and 69 bp [11,17]. The *attP* site in SV1 is therefore likely to be located between nucleotides 20504 and 20545 and is downstream of the integrase gene, *g27*.

**Figure 1:**
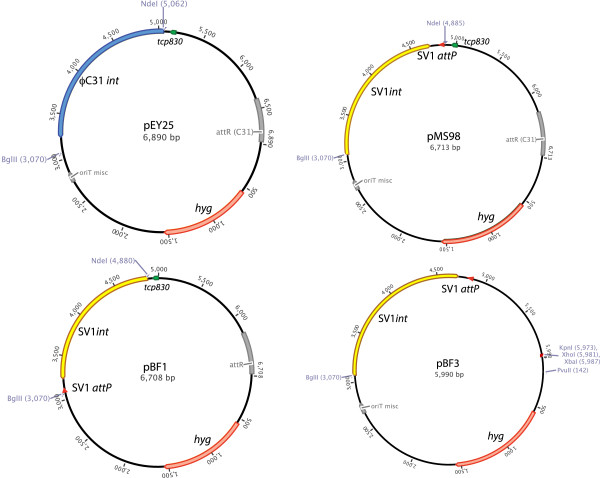
**Plasmids constructed during the course of this work.** pMS98 and pBF1 are derivatives of the plasmid pEY25, in which the φC31 *int* gene (blue arrow) is replaced with the SV1 *g27* (encoding SV1 integrase; yellow arrow) and *attP* (red arrow) in orientations in which the SV1 *g27* genes is being expressed from the *tcp830* promoter (pBF1) or is orientated opposite to the *tcp830* promoter (pMS98). pBF3 is a derivative of pMS98 in which the *tcp830* promoter and the φC31 *attR* sites have been removed. The hygromycin resistance gene is represented by the pale red arrow and the *tcp830* promoter by the green arrow. *oriT* is in grey.

As the SV1 *g27* is likely to be expressed as part of an operon, we decided to test its integrating properties by swapping the ϕC31 *int* ORF expressed from the *tcp830* promoter in pEY25 for the SV1 *g27/attP* locus (Figure 1). Primers were designed to clone the SV1 *g27/attP* locus 20487 to 22295 comprising the putative *attP* site and the *g27* ORF in both orientations downstream of the *tcp830* promoter in pEY25, to generate pMS98 and pBF1 (Figure 1). Both plasmids were introduced into *E. coli* ET12567 (pUZ8002) and used in conjugation reactions with *S. coelicolor* J1929. Surprisingly the numbers of exconjugants were not significantly different for pBF1 (in which the SV1 *g27* is co-directional with the *tcp830* promoter) and pMS98 (in which the SV1 *g27* is oriented towards *tcp830*) and this occurred with or without addition of anhydrotetracycline (Table 1). In fact exconjugants containing pBF1 tended to overproduce the two pigments actinorhodin and undecylprodigiosin indicating a possible stress response, perhaps due to overexpression of SV1 integrase. We assume that there are fortuitous sequences upstream of the *g27* gene in pMS98 that resemble both promoter and ribosome binding sites for integrase expression after conjugation to *Streptomyces*. The low frequency of exconjugants from *E. coli* containing pEY25 in this experiment is likely to be due to the absence of the ϕC31 *attP* site (Figure 1).

**Table 1:** **Conjugation frequnecies of various integrating plasmids into *S. coelicolor *** **J1929**

***E. coli *** **ET12567(pUZ8002) donor containing:**	**Origin of integrase and *attP***	**Hygromycin resistant exconjugants/10**^**8 **^**spores**
pMS98	SV1	2.4 × 10^6^
pBF1	SV1	1.1 × 10^7^
pBF1*	SV1	9.9 × 10^6^
pBF3	SV1	1.4 × 10^7^
pEY25	φC31 (*int* only)	9 x10^3^

*The SV1 integrase was induced with 1 μg/ml anhydrotetracycline.

**Figure 2:**
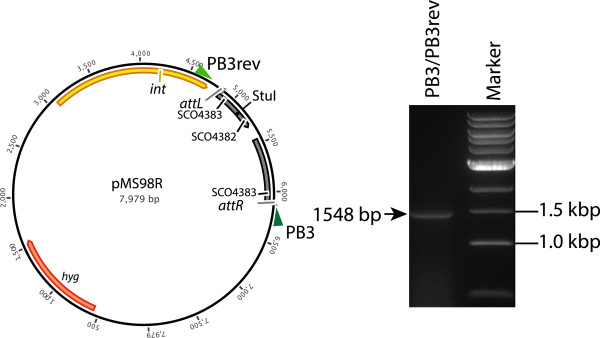
**Rescue of the integrated plasmid and determination of the sequence of *attB*.** The structure of the rescued plasmid, pMS98R, by digestion of genomic DNA from an S. coelicolor J1929 pMS98 exconjugant with StuI and self-ligation. The two primers PB3 and PB3rev were used to amplify the DNA reading out from the *attL* and *attR* sites produced on integration of pMS98 into the *attB* site. The PCR amplified DNA generated was separated by electrophoresis on a 0.8% agarose gel. The size of the band obtained is in agreement with the predicted 1548 bp fragment, after performing the manipulations *in silico* using the published *S. coelicolor* genome sequence [19].

**Figure 3:**
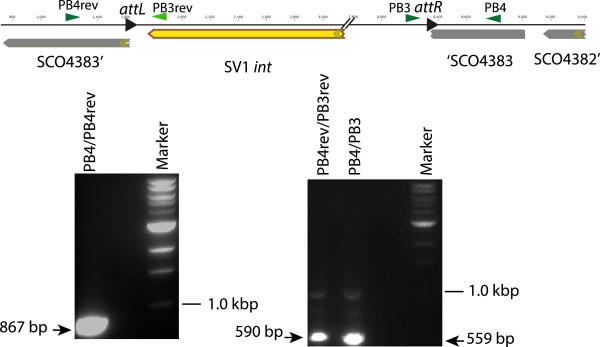
**Validation of the position of the integrated plasmid, pMS98, in the *S. coelicolor *genome.** Primers PB4 and PB4rev were designed to flank the SV1 *attB* site and were predicted to amplify a 867 bp fragment as shown in the agarose gel depicted on the left. Using pairs of primers PB4rev with PB3rev and PB3 with PB4, the *attL* and *attR* sites were amplified from *S. coelicolor* J1929:pMS98 genomic DNA. Fragments of the predicted sizes were obtained as depicted in the agarose gel (right).

**Figure 4:**
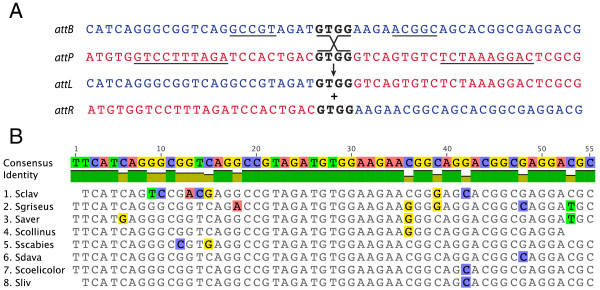
**Sequences of the *attB, attP, attL *and *attR *sites used by SV1 integrase.** Panel **A**. The crossover site occurs within the 4 bp sequence in black and the positions of the inverted repeats are underlined in the *attP* and *attB* sites. Panel **B**. The results of a BLAST search for the SV1 *attB* site in *Streptomyces* genomes deposited in the nucleotide sequence database. The default settings did not identify an SV1 *attB* site from *S. venezuelae* or *S. albus*.

To validate the integration of the plasmids via SV1 *g27/attP* site into the *S. coelicolor* genome, we have identified the integration site of SV1 phage using an inverse PCR technique. Genomic DNA from an exconjugant of *S. coelicolor* J1929 containing the integrated pMS98 was cut with a restriction enzyme for which there is no recognition site within pMS98 and self-ligated. Primers reading outwards from the SV1 plasmid into the *S. coelicolor J1929* genome were then used to generate a PCR product (Figure 2). The plasmid pMS98 was found to have integrated into SCO4383 encoding a putative 4-Coumarate-CoA Ligase, a key enzyme in the phenylpropanoid pathway that, at least in plants, is important in secondary metabolism pathways for flavonoids and monolignols [18]. Based on the DNA sequence of this PCR product, we constructed two further primers against the integration region (SC04383) to amplify *attB*, *attR*, and *attL* (using *S. coelicolor* DNA, and *S. coelicolor* J1929:pMS98 genomic DNA as templates) (Figure 3). The resulting DNA sequences confirmed that the SV1 *attB* site is within SCO4383 and the *attP* site is located, as predicted, downstream of the SV1 *g27* gene (Figure 4). The *attP* and the *attB* have similar features to those observed in the other serine integrase attachment sites; they both contain inverted repeats but these are different in the *attB* and *attP* sequences, and there is an identical sequence of 4 bp in the centre of both substrates where the recombination occurs (Figure 4) [11,12]

Plasmid pMS98 was modified to remove unnecessary DNA, the *attR* site from ϕC31, and the *tcp830* promoter to generate pBF3 (Figure 1). This novel integration vector has unique XhoI, XbaI, KpnI and PvuII sites for cloning. To test whether pBF3 could integrate into a range of *Streptomyces* genomes it was introduced by conjugation into *S. coelicolor, S. lividans, S. venezuelae, S. avermitilis* and *S. albus*. Conjugation frequencies of pBF3 into *S. coelicolor* and *S. lividans* were reminiscent of those obtained with ϕBT1 and ϕC31 integration plasmids; the numbers of hygromycin resistant exconjugants for *S. coelicolor J1929, S. coelicolor M512,* and *S. lividans TK24* were greater than 1 × 10^5^ (Table 2), and the integrations were stable after two rounds of sporulation without selection (see below). When *S. venezuelae* was used as the recipient, the hygromycin resistant exconjugants were, although numerous, very small and the integrations were not stable as after two rounds of sporulation, hygromycin resistance was lost. A BLAST search using the SV1 *attB* site from *S. coelicolor* revealed that highly similar sequences were indeed present in *S. lividans* and *S. avermilitis* but no homologous sequences were found in *S. albus* or *S. venezuelae*. Despite the presence of a putative *attB* site for SV1 in *S. avermitilis* the frequency of conjugation and integration of pBF3 was very low (Table 2).

**Table 2:** **Conjugation frequencies per 10**^**8 **^**spores of integrating plasmids into *Streptomyces *** **species**

***Streptomyces *** **recipient:**	***E. coli *** **donor, ET12567 (pUZ8002) containing plasmids:**			
	**pBF3 (SV1 *g27/attP*)**	**pMS82 (**ϕ**BT1 *int/attP*)**	**pSET152 (**φ**C31 *int/attP*)**	**pRT801 (**φ**BT1 *int/attP*)**
*S. coelicolor* J1929	2.5 × 10^6^	3.6 × 10^6^	6 × 10^5^	ND^1^
*S. coelicolor* M512	1.7 × 10^7^	6 × 10^7^	6 × 10^6^	ND
*S. lividans*	1.4 × 10^5^	9.5 × 10^5^	2.3 × 10^5^	ND
*S. venezuelae* 10712	3.3 × 10^4^	1.7 × 10^7^	9.8 x 10^4^	ND
*S. avermitilis*	40	3 × 10^5^	1.8 x 10^3^	ND
*S. albus* J1074	4.6 × 10^2^	5 × 10^5^	1 x 10^3^	ND
*S. coelicolor* M512: pBF3	-	-	4 x 10^6^	2.8 x 10^5^

^1^Not Done.

The sequences of the SV1 *attB* and *attP* sites are distinct from the recombination sites for the other known phage integrases. We showed previously that integrating vectors derived using integrases from ϕC31 and ϕBT1 do not interfere with each other with respect to the frequency of integration or their stability [12]. We therefore tested whether the integration frequencies of ϕC31 or ϕBT1 derived integrating vectors were affected if the recipient already contained pBF3 integrated at the SV1 *attB* site. Conjugations were performed using *E. coli* donors containing either pSET152 (encoding ϕC31 *int/attP*) or pRT801 (encoding ϕBT1 *int/attP*), both plasmids conferring apramycin resistance, and *S. coelicolor* M512 containing pBF3 as recipient. Selection was for both hygromycin and apramycin. There was no great reduction in the conjugation frequency compared with the use of plasmid-free *S. coelicolor* M512 as a recipient (Table 2). SV1 vectors can therefore be used in combination without interference with ϕC31 and ϕBT1 derived vectors.

**Figure 5:**
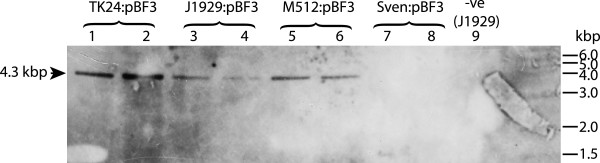
**Southern blot to demonstrate the presence of the predicted hygromycin gene fragment in *S. coelicolor *and *S. lividans *exconjugants.** Two independent exconjugants derived from an *E. coli* donor containing pBF3 and *S. coelicolor*, *S. lividans* and *S. venezuelae* as recipients were initially selected using the hygromycin resistance marker on pBF3 but then subsequently maintained without selection. Genomic DNA was then prepared from each line. The *hyg* gene was detected in *S. coelicolor*:pBF3 and *S. lividans*:pBF3 lines but not in the *S. venezuelae* lines indicating that pBF3 is not stably maintined in *S. venezuelae*.

Hygromycin resistant colonies obtained after conjugation of *E. coli* containing pBF3 with *S. coelicolor J1929, S. coelicolor M512*, *S. lividans TK24 and S. venezuelae* were allowed to sporulate and were subcultured twice on medium without selection. Genomic DNA was isolated and analysed by Southern blotting (Figure 5). The expected 4.3 kbp band, indicative of integrated pBF3, was observed in the DNA from *S. coelicolor* and *S. lividans* exconjugants. The absence of the 4.3 kbp band from the *S. venezuelae* genomic DNA indicated that pBF3 did not persist in this strain and was lost. Stable hygromycin resistant *S. venezuelae* exconjugants were obtained with the ϕBT1 and ϕC31 derived vectors (pMS82 and ϕC31, respectively; Table 2). Paradoxically a lysogen of SV1 in *S. venezuelae* is perfectly stable and grows like the non-lysogen. We deduce that the interruption in SCO4383 caused by the integrating plasmid is toxic, but the toxicity is ameliorated by a prophage-encoded gene or by an unknown *cis* effect within the integrated prophage. Notably SV1 does not encode a homologue to SCO4383, or fragments of SCO4383 that could compensate for its truncation by integration of SV1 derived integrating vectors.

## Conclusions

The activity of a novel phage integration system from bacteriophage SV1 has been demonstrated in *S. coelicolor* and *S. lividans* and the *attP* and *attB* sites identified. We believe that the new integrating vector pBF3 will be of use in the genetic manipulation of these and other *Streptomyces* strains. More generally the characterization of a new integrase and its substrates will provide biologists with new tools for DNA assembly in the genomes of a wide range of microorganisms and other model organisms.

## Methods

### Bacterial strains and culture

*E. coli* strain DH5α was used for plasmid construction. *E. coli* strain ET12567 (pUZ8002) is a methylation-defective strain (*dam-13: Tn9 dcm-6 hsdM*) and was used as the conjugation donor in plasmid conjugations from *E. coli* to *Streptomyces*[20].

Six *Streptomyces* strains were used as recipients for intergeneric conjugation: *Streptomyces coelicolor* J1929 (contains Δ*pglY* conferring sensitivity to ϕC31 and ϕBT1; [21]) *Streptomyces coelicolor* M512 (*ΔredD ΔactII-ORF4* SCP1^−^ SCP2^−^ Pgl^+^) [22], *Streptomyces avermitilis MA-4680*[23], *Streptomyces venezuelae 10712*[24], *Streptomyces albus J1074*[25], *Streptomyces lividans TK24* (*str-6* SLP2^−^, SLP3^−^)[26].

The *E. coli* strains DH5α [27] and ET12567(pUZ8002)[20,26] were grown in Luria-Bertani broth (LB) or on LB agar at 37°C. *Streptomyces* strains were grown in Soya Mannitol (SM) agar at 30°C for routine maintenance [26]. Conjugations were performed on SM containing 10 mM MgCl_2_ and Yeast extract malt extract medium was used for the preparation of genomic DNA [26]. Antibiotic concentrations for *E. coli* were 150 μg/ml hygromycin, 50 μg/ml apramycin, 50 μg/ml kanamycin, 25 μg/ml chloramphenicol and 100 μg/ml hygromycin, 50 μg/ml apramycin and 25 μg/ml nalidixic acid for selection with *Streptomyces*.

### DNA manipulation

Plasmids preparations, *E. coli* transformations, DNA digestion by restriction enzymes, DNA fragment isolation and purification, and gel electrophoresis were carried out according to Sambrook *et al.*[27]. In-Fusion® cloning (Clontech®) was generally used for joining DNA fragments. DNA preparation from *Streptomyces* was performed following the *Streptomyces* manual [26].

Southern blotting was performed, according to the manufacturer^’^s instructions, on Hybond-N nylon membrane (Amersham) using a fragment of DNA derived from the hygromycin resistant gene as the probe. The AlkPhos Direct Labeling and Detection System with CDP-Star kit (Amersham) was used for detection. 1 μg of *Nru*I (New England Biolabs) digested genomic DNA was loaded onto a 0.8% agarose gel in TBE buffer and electrophoreses overnight prior to capillary blotting.

Polymerase Chain Reaction (PCR) was carried out using Phusion® High-Fidelity DNA Polymerase (New England Biolabs) according to the manufacturer^’^s instructions.

### Plasmid constructions

pEY25 is a derivative of pAV11, an integration vector that encodes the ϕBT1 *int/attP* locus and the anhydrotetracycline inducible promoter, *tcp830*. To generate pEY25 the ϕBT1 *int* gene was deleted and the ϕC31 *int* gene was placed under the control of the *tcp830* promoter. pMS98 was constructed by PCR amplification of SV1 *g27/attP* locus using primers MS409 (5′ GCTTCATATGAAACGAGACCTACCAAG) and MS410 (5′CGTTAGATCTTCGCGCTCCGATGTGGTC) and In-Fusion® cloning into pEY25 cut with NdeI and BglII to replace the ϕC31 *int* gene. pBF1 was constructed in the same way but using primers PBF1for (5′ AAGGAGATATACATATGAAACGAGACCTACCAAGC- 3′) and PBF1rev (5′ CCATGAGCCAAGATCTTCGCGCTCCGATGTGGTCC- 3′). pBF3 was constructed as follows to remove unnecessary elements of pBF1: pBF1 was first cut with *Avr*II, and *Acc*65I, and the ends filled in with DNA Polymerase I, Large (Klenow) Fragment (New England Biolabs) to generate blunt ends for ligation. This blunt ended fragment was then self-ligated using Quick ligase enzyme (New England Biolabs) to produce pBF2. To remove the *tcp830* promoter, pBF2 was digested with *Nde*I, and *Ase*I and the 5985 bp fragment was self-ligated to form pBF3.

### Inverse PCR

Inverse PCR was performed to identify the integration site of SV1 within *Streptomyces coelicolor* J1929. This procedure is designed for amplifying anonymous flanking genomic DNA regions. Genomic DNA was prepared from a strain containing the integrated plasmid, pMS98, digested with an enzyme that does not cut within the plasmid (StuI) and then ligation of DNA under dilute DNA conditions to favour circularization. Finally, PCR amplification was performed using oligonucleotides PB3 for (5′ GTACGTCGGAGGTCTAGAGA) and PB3rev (5′ GCAGCTTCGAGTTTCATCCCG) that prime DNA synthesis from the known sequence within pMS98. To confirm the SV1 integration site, primers PB4 for (5′ CACAGCCCCAACACCGTC) and PB4 rev (5′ -GTCGGTGAGGGAGACGATG) were designed to amplify the potential SV1 *attB* from the *S. coelicolor* J1929 DNA. These primers were also used with PB3 and PB3rev to amplify the potential *attR, attL* from the exconjugants *S. coelicolor* J1929 pMS98 DNA.

## Competing interests

The authors declare that they have no competing interests.

## Authors’ contributions

BF designed and performed the experiments and helped to write the paper. EY and GT designed and performed experiments. MCMS designed experiments, raised funding and helped to write the paper. All authors read and approved the final manuscript.
